# The Effect of Self-Sacrificial Leadership on Employees’ Organisational Citizenship Behaviour for the Environment: A Moderated Mediation Model

**DOI:** 10.3390/ijerph19127450

**Published:** 2022-06-17

**Authors:** Yiying Qu, Zhenting Xu, Hong Sun, Qingsheng Li

**Affiliations:** 1 Business School, East China University of Political Science and Law, Shanghai 200042, China; sdlzqyy@hotmail.com (Y.Q.); sunhong_ifrs@163.com (H.S.); 2 Business School, Qingdao University of Technology, Qingdao 266525, China; 3 School of Business, Linyi University, Linyi 276000, China; liqingsheng@lyu.edu.cn

**Keywords:** self-sacrificial leadership, corporate social responsibility, pro-environmental organisational climate, organisational citizenship behaviour for the environment

## Abstract

In the face of increasing environmental pressures, environmentally friendly behaviour can help companies achieve truly sustainable growth. The issue of how to promote environmental behaviour among employees is a new challenge for leaders. However, studies do not systematically reveal the mechanisms of the effects of self-sacrificial leadership on employees’ organisational citizenship behaviour for the environment (OCBE). Based on social learning theory and the attitude–behaviour–context model, we investigated the impact of self-sacrificial leadership on employees’ OCBE by focusing on the mediating role of the corporate social responsibility (CSR) as perceived by employees, and the moderating role of the pro-environmental organisational climate (PEOC). The results of a field survey of 461 employees (small- and medium-sized enterprises) in China indicate that self-sacrificial leadership was positively related to employees’ OCBE; this relationship was partially mediated by employees’ perception of CSR. Moreover, PEOC strengthened the effect of employees’ perceived CSR on OCBE, and the mediating effect of employees’ perceived CSR on the relationship between self-sacrificial leadership and OCBE. Our findings not only help scholars understand the mechanism of the effect of self-sacrificial leadership on employees’ OCBE, but also provide insights for recommending integrated management models, social responsibility, and environmental protection.

## 1. Introduction

Faced with the spread of COVID-19, extreme weather events, and economic turbulence, environmental protection and resource conservation have become important goals so that China can achieve sustainable development. As the primary mover in economic development, enterprises have assumed responsibility for environmental protection and resource conservation, implementing a series of formal regulations and measures, such as environmental industry codes and enterprise environmental regulations [1,2]. To effectively complement the environmental behaviours required by formal rules and regulations, Boiral et al. [3] developed the concept of organisational citizenship behaviour for the environment (OCBE), defining it as the spontaneous and proactive environmental behaviour of individual employees in enterprises that both contributes to the implementation of environmental initiatives facilitated by the company, and enhances its environmental performance. OCBE has become an important research issue in the Chinese context [2,4]. Environmental citizenship behaviours, such as employees voluntarily suggesting reduced energy consumption, separating waste, and using environmentally friendly items, not only contribute to the organisation’s sustainable development, but also promote ecological environmental civilisation. Thus, we explore the important theoretical and practical significance of the influence factor of OCBE.

According to social learning theory, individuals learn through observation of role models to provide specific guidance for their behaviour [5]. Organisational leaders have provided an important imitation model for employees to use when adjusting their behaviours [6]. In other words, leadership has played a critical role in shaping employees’ environmental behaviours [6,7,8]. Accordingly, our research purpose is to explore the influence mechanism of OCBE from the perspective of leadership. Self-sacrificial leadership has attracted the attention of theoretical scholars during crises caused by epidemics and environmental problems [9,10]. Self-sacrificial leadership refers to leaders who voluntarily forgo their personal interests in an effort to maximise the collective welfare [11]. The self-sacrificial leader acts in a manner that demonstrates a high level of ethical responsibilities and duties [9,11], which encourages employees to see them as role models worthy of imitation. As suggested by social learning theory, employees are more likely to learn to “put everything on the line” for others from the leader, high in self-sacrifice, which, in turn, motivates extra-role behaviour, such as organisational citizenship behaviour (OCB) [10,12]. Whereas the research has explained the role of self-sacrificial leadership in OCB, it has neglected the empirical evidence of the influence of self-sacrificial leadership on OCBE. As specific types of OCB [13], both OCBE and OCB emphasise employee-initiated behaviours within organisations that are voluntary and not required by formal reward systems or rules and regulations, and that contribute to organisational effectiveness [3]. However, unlike OCB, with its two dimensions of organisational and interpersonal relationships, OCBE involves environmentally oriented, proactive individual behaviour that complements formal corporate environmental practices [3,14]. For this reason, we examine the effect of self-sacrificial leadership on OCBE.

Why does self-sacrificial leadership enhance employees’ OCBE? To answer this question, we uncover the mediating mechanism of this phenomenon. We explain the relationship between self-sacrificial leadership and employees’ OCBE based on social learning theory, which suggests that employees learn from and imitate leaders’ behaviours, which, in turn, affects their attitudes and behaviours [5,15]. We argue that employees are motivated to engage in OCBE by perceiving the CSR displayed by leaders through the self-sacrificial behaviour in favour of the collective and their followers. Accordingly, we empirically test the mediating effect of employees’ perceived CSR on the relationship between self-sacrificial leadership and OCBE.

The attitude–behaviour–context model suggests that attitudes do not necessarily lead to a behaviour that is difficult or costly to achieve, but instead the process is also influenced by the moderating effect of situational factors [16,17]. Not all employees transform their perceptions of CSR into practical OCBE, because the motivation for their behaviour may require boundary conditions. Therefore, we also explore the moderating role of organisational situational factors in the process through which self-sacrificial leaders influence employees’ OCBE. PEOC, defined as employees’ shared perception of organisational policies or norms that support environmental sustainability in the workplace, has been shown to be positively related to environmental behaviours [1]. Therefore, we identify PEOC as a moderator, examining the moderating effects of PEOC on the relationship between perceived CSR and self-sacrificial leadership with employees’ OCBE.

**Figure 1:**
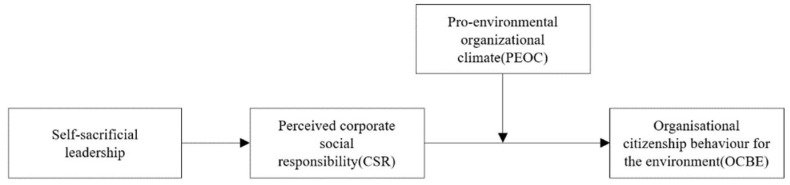
The conceptual model.

We seek to make three theoretical contributions. First, we extend both the leadership and OCBE literature by theoretically building and empirically examining a model to link self-sacrificial leadership and employees’ OCBE in the Chinese context, which can direct scholars to further discuss the effect of leaders’ behaviour on OCBE, and guide managers to understand the environmental effect of self-sacrificial leadership. Second, we explore the mediating effects of employees’ perceived CSR on the self-sacrificial leadership–OCBE relationship, thereby expanding further knowledge of how self-sacrificial leadership encourages employees to achieve OCBE. Third, we demonstrate the moderating effect of PEOC to test a comprehensive picture of when perceived CSR and its mediating role will be enacted and yield higher OCBE among employees. We present the conceptual model in Figure 1.

## 2. Theories and Hypotheses

### 2.1. Self-Sacrificial Leadership and OCBE

OCBE is defined as “individual and discretionary social behaviours that are not explicitly recognized by the formal reward system and that contribute to a more effective environmental management by organizations” [18] (p. 223). Similar to OCB, OCBE emphasises employee-initiated environmental behaviours within an organisation that are voluntary and not required by the rules and regulations of any formal reward system. Employees’ OCBE can effectively complement an organisation’s formal environmental system and reflect the diffusion of environmental awareness and behaviours from daily life to the organisation’s internal environment [19]. Studies have demonstrated that leadership is an important predictor of OCBE [4,20]. These studies have primarily focused on the relationship among ethical leadership [15,21,22], pro-environmental transformational leadership [2], and OCBE; however, they have ignored the relationship between self-sacrificial leadership and employee OCBE. Given the importance of leadership in Chinese collective-oriented contexts, self-sacrificial leadership brings positive outcomes to organisations [9], and its modelling of high moral standards and group-oriented behaviours may positively influence employees’ OCBE.

Self-sacrificial leadership is referred to as “the total/partial abandonment, and/or permanent/temporary postponement of personal interests, privileges, and welfare in the (a) division of labour, (b) distribution of rewards, and/or (c) exercise of power” [11] (p. 479). Self-sacrificial leadership involves volunteering for the benefit of the collective at the expense of one’s personal resources [9,23]. The essential characteristics of self-sacrificial leaders are manifested in proactive responsibility, a collectivist orientation, and service to organisations and employees, all of which positively influence employee reciprocal behaviour [24], pro-social behaviour [23], and OCB [12,23]. For the reasons set forth below, we argue that self-sacrificial leadership influences OCBE.

First, social learning theory suggests that employees who see their leaders as role models, imitating and learning from their virtues, increase their moral responsibility and exhibit a variety of positive behaviours [25,26]. Leaders become role models for employees by demonstrating high ethical standards and maximising the collective welfare at the expense of their own, such as by voluntarily surrendering their power to benefit the collective, thus motivating employees to learn through imitation. Motivated by self-sacrificial leaders’ high ethical standards and sense of responsibility, employees may consider the potential benefits they can provide to the organisation by engaging in pro-environmental behaviours. The OCBE concept includes a strong ethical component [27]; in other words, employees who engage in OCBE learn from their leaders’ organisation-oriented self-sacrifice. Recent studies have provided evidence that leaders’ ethical behaviour promotes employees’ OCBE [3,22].

Second, unlike ethical, transformational, and charismatic leaders, one important characteristic of self-sacrificial leadership is the leader’s self-sacrificial behaviour on behalf of the collective, indicating that organisational goals and welfare are rendered both salient to employees and worthy of their individual, dedicated efforts [28]. Employees are motivated to exceed the obligations of their role to volunteer to engage in environmental protection behaviour in the workplace, such as separating waste, using environmentally friendly products, and recycling old items. Although such behaviour is not required by formal organisational systems and cannot be formally rewarded [13], employees imitate their leaders’ principles of doing what is needed for others and the organisation, thus demonstrating environmental citizenship behaviours. In support of our argument, research has revealed that self-sacrificial leadership is positively related to employees’ OCB [12,29,30], and the concepts underlying OCB and OCBE emphasise employees’ pro-organisational out-role behaviour [21,22]. Thus, we propose the following hypothesis:

**Hypothesis** **1** **(H1).** *Self-sacrificial leadership is positively related to employees’ OCBE*.

### 2.2. Employees’ Perceived CSR as a Mediator

CSR is conceptualised as “corporate behaviours which aim to affect stakeholders positively and go beyond its economic interest” [31] (p. 191). In terms of employees as stakeholders, employees’ perceived CSR has been referred to as a subjective perception of CSR [31,32,33]. Whereas CSR is an organisational-level concept, employees’ perceived CSR is an individual-level concept from the employee’s perspective. Employees evaluate the social responsibility of their leader or organisation as a concern for the collective welfare or employees’ interests [31]. Social learning theory proposes that leaders as role models, who are learnt from and imitated by employees, often influence employees’ perception and behaviours [5]. It is more likely that employees perceive the organisation’s social responsibility based on the leader’s high standard of moral behaviours [34,35]. Self-sacrificial leaders with superb charisma and dedication pay more attention to the welfare of the collective or others than to their own, becoming high-level ethical role models, causing employees to perceive CSR, and stimulating positive altruistic behaviours [36], such as OCBE. When employees perceive that organisations act in a manner that is consistent with social responsibility, they demonstrate more altruism and responsibility, and people with a good sense of social responsibility are more likely to engage in pro-social behaviours, care for the environment and conserve resources, and adopt environmentally friendly citizenship behaviour. Thus, we suggest that employees’ perceived CSR may affect employees’ OCBE.

First, self-sacrificial leadership may facilitate employees’ perception of social responsibility. According to social learning theory, people observe, learn, and imitate their role models’ behaviours [5,8], which suggests that employees can imitate their self-sacrificial leaders’ behaviours. Self-sacrificial leadership demonstrates the moral quality of temporarily or permanently forgoing one’s own interests in favour of the collective, for example, by taking responsibility, putting the interests of the collective first, caring for employees, and assisting in career development [11]. The moral standards of self-sacrificing behaviours are perceived as CSR by employees. Thus, employees believe that leaders and their organisations go the extra mile for them or other stakeholders [34], including by working to protect the environment. Furthermore, they are likely to learn from their leaders’ self-sacrificial behaviours, and are willing to actively take responsibility and make an effort for the sake of both the collective and others. Thus, self-sacrificial leadership can facilitate the CSR perceived by employees.

Second, employees’ perceived CSR may positively affect their OCBE, which is framed as a spontaneous extra-role behaviour [13]. CSR perceived by employees who believe their organisation will do what is morally right for the sake of the collective is particularly important in improving those employees’ OCBE. According to social learning theory, if employees recognise that corporate behaviours are praiseworthy, they tend to imitate altruistic behaviours in their own work. The more CSR is recognised by employees, the more social responsibility-driven citizenship behaviours they engage in, such as suggesting improved corporate environmental policies, and sorting and recycling office waste. Recent studies have provided evidence that CSR is related to both OCB [35,36,37] and green behaviour [38]. Integrating the above arguments with those supporting the positive link between self-sacrificial leadership and employees’ perceived CSR discussed earlier, we propose the following hypothesis:

**Hypothesis** **2** **(H2).** *Employees’ perceived CSR mediates the relationship between self-sacrificial leadership and employees’ OCBE*.

### 2.3. PEOC as a Moderator

The attitude–behaviour–scenario model based on field theory illustrates that employees’ behavioural decisions are influenced not only by their own attitudes [16], but also by organisational situational factors [17]. Organisational situational factors affect employee behaviours by creating the organisational climate [39]. Organisational climate is considered an important environmental situational factor affecting employee behaviour [40,41,42]. Employees integrate their own cognition and situational factors to make decisions. Therefore, we introduce PEOC, as a specific context of organisational climate in terms of environmental protection [43], to explain the moderating effect of differences in environmental climate in organisations on the impact of employees’ perceived CSR on employees’ OCBE.

PEOC is characterised as employees’ common perception of environmental sustainability policies, norms, and practices in organisations, including support for not only an organisation’s formal green system, but also its environmental requirements and values [43]. We find that PEOC is the specific organisational climate formed by a series of pro-environmental policies that promotes positive environmental values to employees, and facilitates the implementation of environmental behaviours. As an environment-oriented climate, PEOC has increased the encouragement of and support for employees’ environmental behaviours, enabling employees to engage in those behaviours, such as saving resources and making environmental suggestions [1,21]. Most scholars have suggested that PEOC, or employees’ perceptions of the pro-environmental facet of the organisational climate (including a range of pro-environmental concepts, procedures, and practices, along with support from their colleagues), can positively influence employee green behaviour [1,39]. We consider PEOC as a moderator that determines the extent to which employees can convert perceived CSR into positive OCBE.

Although individuals with a strong sense of social responsibility may exhibit more altruistic or citizenship behaviours [36], not all such individuals necessarily exhibit pro-environmental behaviours. Employees who have a sense of CSR need to be in an environmentally friendly atmosphere to implement individual behaviour geared towards caring for the environment and saving resources [34,35]. The pro-environmental climate represents the extent to which organisations pay attention to environmental issues, adopt formal green policies in different business processes, and support sustainable activities. Such pro-environmental norms motivate employees to implement their pro-environmental intentions (i.e., to translate their sense of social responsibility to the company into pro-environmental behaviour, such as environmental citizenship). In the workplace, employees exposed to a strong PEOC in which they can facilitate the organisation’s daily work practices to support environmental goals and values are more likely to perceive that their organisation encourages them to accomplish their tasks in an eco-friendly way, facilitating environmental citizenship behaviour. Therefore, employees perceive the organisation as pro-environmental, and strongly identify with the organisation to undertake CSR to achieve its sustainable development. In contrast, employees working in a poor environmental climate may find it difficult to perceive the organisation’s environmental strategy, and thus, they are more likely to engage in more organisation- or interpersonal-oriented citizenship behaviour motivated by high CSR [44], but not to engage in environmental behaviour. Thus, PEOC catalyses eco-friendly behaviours facilitated by employees with CSR, strengthening the relationship between employees’ perceived CSR and OCBE. Therefore, we propose the following hypothesis:

**Hypothesis** **3** **(H3).** *PEOC positively moderates the relationship between employees’ perceived CSR and OCBE such that the relationship is stronger for employees with higher levels of PEOC*.

Considering H2 and H3 and their related studies, we propose that by strengthening the positive relationship between employees’ perceived CSR and OCBE, high PEOC will strengthen the indirect relationship between self-sacrificial leadership and employees’ OCBE via perceived CSR. When the PEOC is high, employees are more likely to adopt the CSR they perceive from self-sacrificial leaders, and are encouraged to engage in more OCBE. In contrast, in the absence of PEOC, even if employees perceive CSR expressed by self-sacrificing leaders, they may not transform their enhanced sense of responsibility into OCBE, instead engaging in other types of performance outcomes. Taken together, we propose the following hypothesis:

**Hypothesis** **4** **(H4).** *PEOC positively moderates the mediating effect of employees’ perceived CSR between self-sacrificial leadership and employees’ OCBE*.

## 3. Methods

### 3.1. Procedures and Sample

The manufacturing industry has a greater impact on the environment in production and management activities, and thus, there is an urgent need for manufacturing enterprises to strengthen technological innovation and adopt clean production processes in China [18,45]. Employees in the manufacturing industry should also take environmental responsibility and implement spontaneous environmental behaviours [46]. In view of this, the survey sample of employees was collected from 16 small- and medium-sized manufacturing enterprises in the Yangtze River Delta region in China. We had good research communication with the target companies, and the survey was supported by both the persons in charge and the respondents. Before collecting the data, we gave the persons in charge advance notice, enabling them to understand the purpose of the study and what we expected them to do in the survey. A web-based research packet, containing cover letters, the questionnaire, and a confidentiality agreement, was sent via a web link. To alleviate concerns about common method bias [47], we follow the extant research to collect data during May–September 2021 in two phases [48,49,50]. In the first wave (time 1), the respondents were asked about their personal backgrounds, self-sacrificial leadership, and PEOC. In the second (time 2), conducted one month after time 1, they were asked about perceived CSR and OCBE. In the first round of data collection, a total of 700 questionnaires were distributed and 582 were collected; in the second round, 492 questionnaires were completed by respondents in the previous round. After eliminating invalid questionnaires, 461 valid questionnaires remained, with a valid response rate of 65.9%.

Of the respondents, 210 (45.6%) were male. The seven age cohorts were below 25 (6.9%), 25–30 (23.9%), 31–35 (27.8%), 36–40 (19.7%), 41–45 (12.1%), 46–50 (6.9%), and over 50 (2.7%) years old; educational attainment was concentrated at the college degree (19.7%) and Bachelor’s degree (56.4%) levels. The average job tenure was 4.15 years (SD = 1.16).

### 3.2. Measures

All of the scales used in this study were originally developed in the West and applied in Chinese settings in previous studies, as shown in Table A1. We obtained the Chinese version of self-sacrificial leadership and OCBE measurements directly from Chinese scholars, and we tested a Chinese version of PEOC and perceived CSR by following Brislin’s [51] translation–back–translation procedure. We invited a management professor to translate the English scale into Chinese, and then invited another management professor to translate it back. In addition, we invited bilingual management professors to check both translations to continuously revise the scale validation. Except for the control variables, all of the core variables were measured by the participants’ responses to questions on a 5-point Likert-type scale ranging from “strongly disagree” to “strongly agree”.

Self-sacrificial leadership: a 5-item scale developed by De Cremer and van Knippenberg [24], and later applied in Chinese settings [9], was used to measure self-sacrificial leadership. One sample item was “My supervisor is willing to make personal sacrifices in the organisation’s interest”. Cronbach’s alpha for this measure was 0.913.

Organisational citizenship behaviour for the environment (OCBE): a 10-item scale developed by Boiral and Paillé [52], and later applied by [20] in China, was used to measure OCBE. One sample item was “I encourage my colleagues to adopt more environmentally conscious behaviours”. Cronbach’s alpha for this measure was 0.934.

Pro-environmental organisational climate (PEOC): a Chinese translation of the four-item scale developed by Norton et al. [43] was used to measure PEOC. Based on the purpose of the study, the four items of this scale corresponded to the organisational level. One sample item was “I encourage my colleagues to adopt more environmentally conscious behaviours”. Cronbach’s alpha for this measure was 0.766.

Employees’ perceived corporate social responsibility (CSR): six items developed by Turkey [33] were used to measure how employees perceive CSR. One sample item was “Encourages its employees to participate in voluntary activities”. Cronbach’s alpha for this measure was 0.798.

Control variables: given our concern about the effects of demographic variables on OCBE [2,19,22], we controlled for employees’ demographic variables, including gender (coded 1 = male and 2 = female), age (coded 1 = below 25 years, 2 = 25–30 years, 3 = 31–35 years, 4 = 36–40 years, 5 = 41–45 years, 6 = 46–50 years, and 7 = more than 50 years), and job tenure (coded 1 = below 1 year, 2 = 1–3 years, 3 = 3–5 years, 4 = 5–10 years, and 5 = more than 10 years). Education levels were hierarchically coded as 1 = high school graduate or below, 2 = college degree, 3 = Bachelor’s degree, and 4 = Master’s degree or above.

### 3.3. Analytical Strategy

We used the SPSS22.0, AMOS22.0, and PROCESS3.3 macro programs for our statistical analysis of the data. First, a set of confirmatory factor analyses (CFAs) was conducted using AMOS22.0 to test the discriminant validity among four latent variables (i.e., self-sacrificial leadership, PEOC, and employees’ perceived CSR and OCBE). Next, descriptive statistical analysis and correlation analysis methods were conducted using SPSS22.0 to examine the relationships between the variables. PROCESS3.3 was used to test the moderated mediation effect in the second stage.

## 4. Results

### 4.1. Confirmatory Factor Analysis

The CFA results are shown in Table 1. The four-factor model achieved a good fit to the data: χ^2^/*df* = 2.069, GFI = 0.914, CFI = 0.931, NFI = 0.922, and RMSEA = 0.061. We then confirmed the discriminant validity of the four-factor model by testing alternative models (three-factor, two-factor, and one-factor models). As suggested by [53], χ^2^/*df* took values between 1 and 5; CFI, GFI, and NFI were greater than 0.9; and RMSEA was less than 0.08, indicating that the model had a good fit. As presented in Table 1, the four-factor model provided a significantly better fit than the alternative models, indicating satisfactory construct validity in the Chinese context.

### 4.2. Common Method Bias

To effectively mitigate the common method bias arising from the same subjects or data sources, we first adopted a two-stage data collection approach while ensuring the authenticity of data acquisition through anonymity and confidentiality measures during the collection process. Second, CFA also showed that the four-factor model had a good fit, and these variables had good discriminant validity, further indicating that the common method bias in this study was not serious. Finally, the total variance explained cumulatively by the four factors extracted from the common method factors was 71.27%, and the maximum value of the variance explained by the factors was 24.98%, which was in accordance with the recommended value of the statistical test, and did not exceed 50% of the total variance. Therefore, common method bias did not affect the relationship between the variables to be tested.

### 4.3. Descriptive Analysis

Table 2 shows the mean, standard deviations, and correlations for the study variables. As the table shows, self-sacrificial leadership was positively related to both employee OCBE (*β* = 0.38, *p* < 0.01) and employees’ perceived CSR (*β* = 0.22, *p* < 0.01). Employees’ perceived CSR was positively related to employee OCBE (*β* = 0.46, *p* < 0.01). Therefore, these results primarily supported the proposed hypotheses, and provided the necessary preconditions for the mediating effect test.

### 4.4. Hypotheses Testing

Main effect: H1 proposes the main effect of self-sacrificial leadership on employee OCBE. Table 3 shows the hierarchical regression results. Self-sacrificial leadership was positively related to employees’ perceived CSR (*β* = 0.20, *p* < 0.01; Model 2) and positively related to employee OCBE (*β* = 0.29, *p* < 0.01; Model 4), thus supporting H1.

Mediating effect: H2 predicts that employees’ perceived CSR mediates the relationship between self-sacrificial leadership and employees’ OCBE. We followed Baron and Kenny’s [54] study to test this mediation. First, the results in Table 3 show that self-sacrificial leadership was positively related to employees’ perceived CSR (*β* = 0.20, *p* < 0.01; Model 2). Second, as indicated in H1, self-sacrificial leadership was significantly and positively related to employees’ OCBE (*β* = 0.29, *p* < 0.01; Model 4). Third, compared to the result shown in Model 2 in Table 3, the coefficient of the relationship between self-sacrificial leadership and employees’ OCBE (*β* = 0.19, *p* < 0.01; Model 5) was diminished when employees’ perceived CSR was included in the model, whereas employees’ perceived CSR was positively related to employees’ OCBE (*β* = 0.48, *p* < 0.01; Model 5). This indicates that employees’ perceived CSR acted as a partial mediator of the relationship between self-sacrificial leadership and employees’ OCBE. Thus, these results initially support H2 [54]. We used a parameter bootstrapping procedure to further verify the mediation relationship [55]. Based on a sample size of 5000, the bootstrap results show that the indirect relationship between self-sacrificial leadership and OCBE via employees’ perceived CSR was significant (*β* = 0.55, 95% CI = (0.03, 0.10)), providing further support for H2.

**Figure 2:**
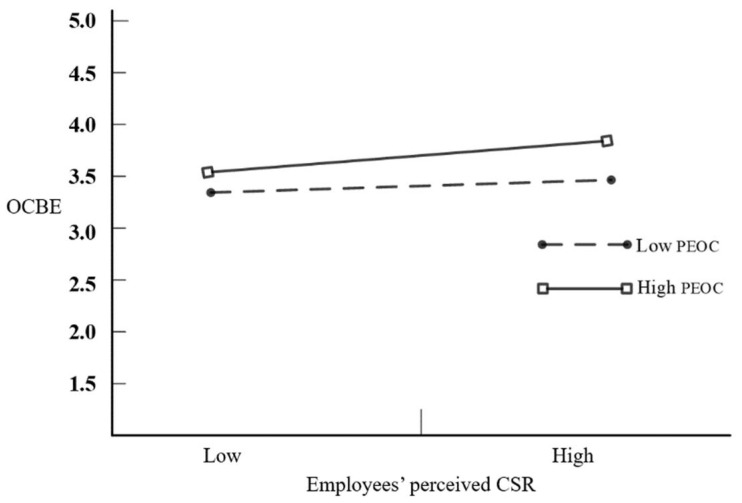
The interaction effect of PEOC on the relationship between employees’ perceived CSR and OCBE.

**Figure 3:**
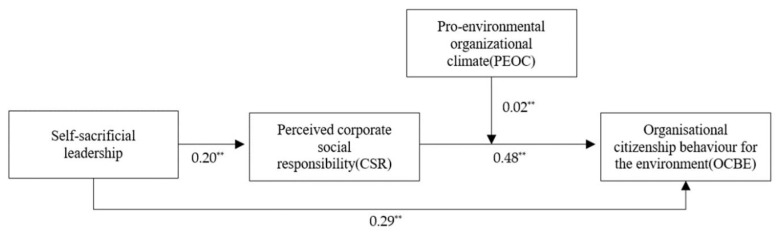
Result of the hypothesised model. Note: ** *p* < 0.01.

Moderated mediation: H3 proposes that PEOC positively moderates the relationship between employees’ perceived CSR and employee OCBE such that the relationship is stronger for employees with higher levels of PEOC. We first standardised employees’ perceived CSR and PEOC to set the interaction term, which was entered into the regression model. As shown in Table 3, the interaction term of employees’ perceived CSR and PEOC was significantly and positively related to employees’ OCBE (*β* = 0.04, *p* < 0.01, Model 7), indicating that PEOC played a positive moderating role in the relationship between employees’ perceived CSR and OCBE. Thus, H3 is supported. The interaction effects are depicted in Figure 2. H4 indicates that PEOC positively moderates the mediating effect of employees’ perceived CSR between self-sacrificial leadership and employees’ OCBE. We conducted PROCESS3.3 to test the second-stage moderated mediation. As shown in Table 4, the value of the index of moderated mediation was 0.0141 (95% CI = (0.0043, 0.0276)), which was significant. Then, we further tested the indirect effect of self-sacrificial leadership on employees’ OCBE via employees’ perceived CSR at different levels of PEOC (one SD below the mean, the mean, and one SD above the mean). As shown in Table 4, the conditional indirect effect of self-sacrificial leadership via employees’ perceived CSR on OCBE was significant at the low level (95% CI = (0.0052, 0.0155)) and at the high level (95% CI = (0.0075, 0.0330)), with the estimated value of the indirect effect increasing from 0.0046 to 0.0187. Therefore, H4 is supported. The results of the hypothesized model are shown in Figure 3.

## 5. Discussion

### 5.1. Theoretical Implications

First, we theoretically establish and empirically examine a conceptual model that integrates leadership with employee OCBE theory. Our results empirically demonstrate, for the first time, that self-sacrificial leadership promotes employees’ OCBE. Among the leadership theories that influence employees’ OCBE, some studies have focused on transformational leadership [8] and ethical leadership [20], but none have examined the relationship between self-sacrificial leadership and employees’ OCBE. In addition, we respond to the call from Lamm et al. [14] to apply a theoretical model to verify the influence of various types of leadership on OCBE, expanding the antecedents of employees’ OCBE. In the field of leadership research, scholars have focused on employee performance-related outcomes of self-sacrificial leadership, such as job performance and employee creativity, ignoring the impact of self-sacrificial leadership on environmental behaviours [12]. Thus, we also answer the call from Daily et al. [13] and Zhang et al. [15] to empirically examine the environmental effect of self-sacrificial leadership. Accordingly, we not only respond to future research directions proposed by theoretical scholars, but also fill research gaps in the literature and enrich the theories of self-sacrificial leadership and OCBE.

Second, based on social learning theory, we find that employees’ perceived CSR partially mediates the relationship between self-sacrificial leadership and OCBE, thus providing a new theoretical framework for understanding the influence process of self-sacrificial leadership on employees’ OCBE. Self-sacrificial leaders are concerned about the collective welfare, and exhibit responsibility and dedication, both of which are perceived by employees as demonstrating the responsibility of the enterprise [9,24]. Employees then tend to exceed their formal job requirements and spontaneously devote themselves to environmental behaviours that are beneficial to the organisation’s image. The results may encourage future scholars to address the impact of leadership on employees’ pro-environment consequences by focusing on the employee ethical factors, including environmental self-accountability [56], prosocial motivation [57], and environmental beliefs [58]. Thus, we provide a clear theoretical framework for the influence of self-sacrificial leadership on employees’ OCBE.

Third, organisational citizenship behaviour theory suggests that the study of employees’ OCBE considers not only individual-level factors, but also situational factors in which individuals are placed so that the situational characteristics of individual factors affecting employees’ OCBE can be clarified [39]. Therefore, we incorporate PEOC into the relationship between self-sacrificial leadership, perceived CSR, and employees’ OCBE. As discussed previously, employees in a strong pro-environmental climate are likely to be motivated to engage in OCBE by perceived CSR from self-sacrificial leadership. This demonstrates the boundary conditions of employees’ OCBE, and enriches PEOC theory to address new features and attributes arising from the interaction of individual and situational factors on OCBE, thus providing new perspectives for future research.

### 5.2. Practical Implications

To achieve sustainable development, enterprises should not only implement formal green policies in management activities, but also encourage employees’ daily environmental citizenship behaviour.

First, our findings show that self-sacrificial leaders are regarded as a key for organisations’ green development in the workplace. Self-sacrificial leaders can set ethical and moral examples and inspire helpfulness on the part of employees in various situations [9,39], which can enhance communication between colleagues, identification with the organisation, and the implementation of environmental protection beliefs. Thus, our work suggests that organisations can cultivate employee CSR and OCBE through enhancing self-sacrificial leadership practice in the Chinese context. When recruiting or selecting leaders, organisations strengthen the moral practices of collective benefits of leaders and guide them to adopt self-sacrificial leadership to undertake responsibility for sustainable development. Besides, more leadership training and development programs can be developed to support self-sacrificial activities to exhibit social responsibility, such as instructing leaders on how to articulate an environmental vision to employees for the collective welfare.

In addition, it may be difficult to implement formal environmental policies in all aspects of enterprise practice, so it is necessary to complement employees’ spontaneous environmental behaviours [59]. Our findings show that employees in a strong pro-environmental climate are more likely to learn CSR from self-sacrificial leaders, thereby engaging in more OCBE. Therefore, while engaging in environmental management practices, leaders who forgo one’s own interests in favour of the collective can encourage employees to better understand CSR, and thus to go beyond their personal interests to protect the environment for the sake of the organization and society.

### 5.3. Limitations and Future Research Directions

Although our study has a certain theoretical and practical value, some limitations remain. First, we adopt a self-reported measurement method that cannot completely avoid common method bias. As the variables measured refer to employees’ perceptions of CSR and leadership, it is difficult to obtain assessments from others. Future research can use colleagues to assess employees’ OCBE and explore the impact of the different perceptions of employees and their colleagues on employees’ OCBE. Second, we use a cross-sectional design, which cannot rigorously test the causal relationship between variables. Future research can use a multiple time-point longitudinal design to enhance the validity of the causal relationship between variables. Third, the findings of this study are limited to the Chinese manufacturing context, and the sampled respondents are from a few enterprises with different pro-environmental degrees. The convenience samples of this study may be biased, which leads to a constraint on the generalisability of the findings [60]. It may be that the variance of the impact of self-sacrificial leadership on employees’ OCBE exists in other industries, and thus, the scope of the study can be further expanded to a multi-industry and even cross-country context. Last, we only examine the moderating effect of PEOC among environmental factors. Future research can investigate the effects of other environmental differences, such as green human resource management [44].

## 6. Conclusions

We integrated research on leadership and pro-environmental behaviour to examine the links between self-sacrificial leadership and employees’ OCBE. Integrating social learning theory and the attitude–behaviour–context model, we extended employees’ perceived CSR to represent a mediating mechanism between self-sacrificial leadership and employee OCBE, and found that PEOC strengthens the effects of employees’ perceived CSR on employee OCBE, and positively moderates the mediating role of perceived CSR between self-sacrificial leadership and employees’ OCBE.

## Figures and Tables

**Table 1:** Confirmatory factor analysis.

Model	χ^2^/*df* (*df*)	GFI	CFI	NFI	RMSEA
**Four-factor model**	2.069 (268)	0.914	0.931	0.922	0.061
**Three-factor model**					
SSL and employees’ perceived CSR were combined	5.697 (271)	0.846	0.860	0.857	0.082
**Two-factor model**					
SSL, and employees’ perceived CSR and PEOC were combined	8.126 (274)	0.721	0.739	0.730	0.103
**One-factor model**	11.247 (275)	0.664	0.682	0.674	0.116

Note: SSL represents self-sacrificial leadership, CSR represents corporate social responsibility, PEOC represents pro-environmental organisational climate, and OCBE represents organisational citizenship behaviour for the environment.

**Table 2:** Means, standard deviations, and correlations.

Variables	Mean	SD	1	2	3	4	5	6	7	8
1. Gender	1.53	0.50								
2. Age	3.53	1.45	0.11							
3. Job tenure	4.15	1.16	0.07	0.18						
4. Education level	2.84	0.80	0.07	0.11	0.08					
5. SSL	3.28	0.77	−0.06	0.05	0.13	0.06	0.913			
6. Employees’ perceived CSR	3.92	0.48	0.06	0.04	0.10	0.02	0.22 **	0.798		
7. PEOC	4.00	0.51	0.02	0.08	0.06	0.04	0.30 **	0.43 **	0.766	
8. OCBE	3.98	0.49	0.03	0.07	0.07	0.04	0.38 **	0.46 **	0.31 **	0.934

Note: N = 461, ** *p* < 0.01. SD = standard deviation. Reliabilities are on the diagonal parentheses. Gender is coded 1 = male and 2 = female; Age is coded 1 = below 25 years, 2 = 25–30 years, 3 = 31–35 years, 4 = 36–40 years, 5 = 41–45 years, 6 = 46–50 years, and 7 = more than 50 years; Job tenure is coded 1 = below 1 year, 2 = 1–3 years, 3 = 3–5 years, 4 = 5–10 years, 5 = more than 10 years; Education level is coded 1 = high school graduate or below, 2 = college degree, 3 = Bachelor’s degree, 4 = Master’s or above. SSL represents self-sacrificial leadership, CSR represents corporate social responsibility, PEOC represents pro-environmental organisational climate, and OCBE represents organisational citizenship behaviour for the environment.

**Table 3:** Hierarchical regression analysis results of variables.

Variables	Perceived CSR		OCBE				
	Model 1	Model 2	Model 3	Model 4	Model 5	Model 6	Model 7
**Control variables**							
Gender	−0.11	−0.09	−0.04	−0.04	0.04	−0.02	−0.02
Age	0.05	0.06	0.00	0.02	−0.01	0.01	0.01
Job tenure	−0.01	−0.00	−0.04	−0.02	−0.02	−0.02	−0.02
Education level	−0.01	−0.00	0.03	0.03	0.04	0.01	0.01
**Independent variables**							
SSL		0.20 **		0.29 **	0.19 **		
**Mediator**							
Employees’ perceived CSR					0.48 **	0.05 **	0.04 **
**Moderator**							
PEOC						0.47 **	0.45 **
**Interaction effect**							
PEOC× Employees’ perceived CSR							0.04 **
R^2^	0.03	0.12	0.01	0.18	0.38	0.83	0.87
ΔR^2^	—	0.09 **	—	0.17 **	0.20 **	^a^ 0.82 **	0.04 **

Note: ** *p* < 0.01. ^a^ compared to model 3. SSL represents self-sacrificial leadership, CSR represents corporate social responsibility, PEOC represents pro-environmental organisational climate, and OCBE represents organisational citizenship behaviour for the environment.

**Table 4:** The moderated mediation effect in the second stage.

	Effect	SE	Boot LLCI	Boot ULCI
Low PEOC	0.0046	0.0052	0.0052	0.0155
Medium PEOC	0.0117	0.0050	0.0034	0.0229
High PEOC	0.0187	0.0064	0.0075	0.0330
	**Index**	**SE**	**Boot LLCI**	**Boot ULCI**
Perceived CSR	0.0141	0.0059	0.0043	0.0276

## Data Availability

The data that support the findings of this study are available from the authors upon reasonable request.

## Appendix A

**Table A1:** Measurement items of key variables.

Variables		Items	Source
Organisational citizenship behaviour for the environment (OCBE)	1	In my work, I weigh the consequences of my actions before doing something that could affect the environment 我在开展工作之前会权衡自己的行为是否有利于环境保护	Boiral and Paillé (2012) [52]; Zhang et al. (2018) [20]
	2	I voluntarily carry out environmental actions and initiatives in my daily work activities 在我的日常工作中, 我自愿开展环保行为和举措	
	3	I make suggestions to my colleagues about ways to protect the environment more effectively, even when it is not my direct responsibility 我会给同事提出保护环境的建议, 即使这不是我的责任	
	4	I actively participate in environmental events organised in and/or by my company 我积极参与企业组织的环保活动	
	5	I stay informed of my company’s environmental initiatives 我保持了解我企业的环保措施	
	6	I undertake environmental actions that contribute positively to the image of my organisation 我从事有利于企业积极形象的环保行动	
	7	I volunteer for projects, endeavours, or events that address environmental issues in my organisation 我自愿承担我企业有关环境问题的各种项目	
	8	I spontaneously give my time to help my colleagues take the environment into account in everything they do at work 我不由自主地帮助同事在工作中考虑环境问题	
	9	I encourage my colleagues to adopt more environmentally conscious behaviour 我鼓励我的同事采用更有环境意识的行为	
	10	I encourage my colleagues to express their ideas and opinions on environmental issues 我鼓励我的同事表达他们对环境问题的想法和意见	
Self-sacrificial leadership	1	My team leader is willing to make personal sacrifices in the team’s interest 我的领导愿意为团队的利益做出个人牺牲	De Cremer and van Knippenberg (2004) [24]; Qu et al. (2021) [9]
	2	My supervisor is willing to stand up for the team members’ interests, even when it is at the expense of his/her own interests 我的领导即使个人利益受损,也愿意维护组织成员利益	
	3	My team leader is always among the first to sacrifice free time, privileges, or comfort if that is important 我的领导在工作中愿意牺牲个人特权、舒适和享受	
	4	I can always count on my supervisor to help me in times of trouble, even if it is at a cost to him/her 在遇到困难的时候,我总是能指望我的领导帮助我,即使帮助我会使领导个人利益受损。	
	5	My supervisor is willing to risk his/her position if he/she believes the goals of the team can be reached that way 我的领导在实现组织目标的过程中,会作出自我牺牲	
Employees’ perceived corporate social responsibility (CSR)	1	Our company encourages its employees to participate in voluntarily activities 我们公司鼓励其员工参加自愿性活动	Turker (2009a) [33]
	2	Our company policies encourage the employees to develop their skills and careers 我们公司的政策鼓励员工发展他们的技能和职业	
	3	The management of our company is primarily concerned with employees’ needs and wants 我们公司的管理层主要关注员工的需求	
	4	Our company implements flexible policies to provide a good work and life balance for its employees 我们公司实施灵活的政策,为员工提供一个良好的工作和生活平衡	
	5	The managerial decisions related with the employees are usually fair 与雇员有关的管理决策通常是公平的	
	6	Our company supports employees who want to acquire additional education 我们公司支持希望获得额外教育的员工	
Pro-environmental organisational climate (PEOC)	1	Our company is worried about its environmental impact 我们公司关注自身对环境的影响	Norton et al. (2014) [43]
	2	Our company is interested in supporting environmental causes 我们公司致力于支持环保事业	
	3	Our company believes it is important to protect the environment 我们公司认为保护环境很重要	
	4	Our company is concerned with becoming more environmentally friendly 我们公司热衷于进一步的环境友好发展	
